# Rethinking cholera response: an orchestration framework for future preparedness in fragile conflict-affected settings, a case study from Sudan, 2025

**DOI:** 10.3389/fpubh.2026.1836433

**Published:** 2026-06-30

**Authors:** Fatima Abdalrahman Ayyad, Ahmad Izzoddeen, Maisoon Elbukhari Ibrahim, Heitham Awadalla

**Affiliations:** 1 Federal Ministry of Health, Khartoum, Sudan; 2 Field Epidemiology Training Program, Federal Ministry of Health, Khartoum, Sudan; 3 Geneva Center for Humanitarian Studies, University of Geneva, Geneva, Switzerland

**Keywords:** cholera response, complex adaptive systems, conflict, health system, Orchestrated Symphony Model, orchestration governance, resilience, Sudan

## Abstract

**Introduction:**

The ongoing conflict in Sudan has intensified the country’s already fragile public health landscape. Since July 2024, Sudan has faced one of the most extensive cholera outbreaks in its modern history, affecting 17 states and 109 localities and resulting in 83,245 reported cases and 2,124 deaths by June 2025. Traditional, hierarchical outbreak response systems have proven insufficient in such a dynamic, volatile environment.

**Methods:**

Using Sudan’s 2024–2025 cholera outbreak as a case study, this paper proposes the *Orchestrated Symphony Model*, an innovative framework that integrates systems thinking and orchestration governance, to design a model for outbreak management in fragile and conflict-affected settings.

**Results:**

The model conceptualizes the Ministry of Health as a conductor leading an orchestra of diverse stakeholders. Each stakeholder represents an instrument section whose coordinated actions create a harmonized and adaptive response. Grounded in systems thinking theory, particularly the Iceberg and Biomatrix models, this framework identifies leverage points for governance, coordination, and resource optimization.

**Conclusion:**

The Orchestrated Symphony Model is proposed as a policy reform and practice improvement framework for cholera outbreak response generally and in conflict-affected settings particularly.

## 1 Introduction

Cholera remains a major global public health threat, causing an estimated 1–4 million cases and 21000–143000 deaths annually, with 47 countries reporting endemic transmission, mainly in Africa and Asia (1). The highest burden is in the developing (Low- and middle-income) driven by many factors including poor sanitation, limited access to clean water and fragile health systems what create ideal conditions for massive outbreaks (2). During the past decade, in the same country category, the pooled cholera case fatality rate was 1.3%, with contaminated water has been the main transmission route underscoring a persistent vulnerability (2). In Africa specifically, the 2025 surge in multiple countries has been linked to political unrest, mass displacement and climate-related factors, severely straining already fragile health systems (3).

The cholera outbreak that emerged in Sudan in July 2024 has unfolded amid severe national instability. The conflict has displaced millions and caused extensive damage to essential water, sanitation, and health infrastructure. As of June 2025, 17 states across the country reported cholera transmission, with 83,245 confirmed cases (attack rate 231.2 per 100,000) and 2,124 deaths (case fatality ratio 2.6%). Khartoum State alone accounted for 27% of total cases and nearly one-fifth of all deaths. These statistics reflect not only an acute public health emergency but also the systemic collapse of essential services that are critical for disease prevention and control (4, 5).

Traditional outbreak response models, largely hierarchical, sectorally divided, and centrally managed, have struggled to cope with the Sudanese context. In conflict-affected environments, a top-down command structure fails to adapt to rapidly shifting realities on the ground, including fluctuating security conditions, constrained access to affected populations, and limited availability of humanitarian partners. As a result, coordination efforts have been fragmented, leading to duplication of effort, inefficient resource allocation, and erosion of community trust.

Sudan’s experience illustrates a broader challenge: how can outbreak response systems in fragile settings move from control to coordination, from fragmentation to flow, and from isolated efforts to systemic orchestration? This paper argues that the solution lies in reframing outbreak governance through a systems-oriented orchestration approach. By integrating systems thinking and orchestration theory, it is possible to redesign coordination into a dynamic, adaptive, and harmonized model capable of functioning in complexity and crisis.

The *Orchestrated Symphony Model* embodies this shift. Drawing on metaphors from music, the framework redefines the Ministry of Health as the “conductor,” leading an “orchestra” of stakeholders—national and local authorities, international agencies, community actors, and technical partners—each representing an instrumental section (surveillance, case management, WASH, logistics, and communication). Through this orchestration, a unified “score” or response plan can be executed with synchronized timing, clear roles, and adaptive coherence (6, 7).

Orchestration governance, as a practical framework, has recently been tested in other low-resource and crisis settings. A scoping review of multi-stakeholder collaboration for infectious disease outbreaks found that trust-building, shared goals, and clear decision-making processes are key determinants of effective coordination—insights that are directly embedded in our Symphony Model’s structure, process, and ethos components (8). Similarly, a review of health system strengthening in fragile and conflict-affected states concluded that “coordinated and integrated responses tailored to the context” are essential, a finding that reinforces our emphasis on adaptive orchestration rather than rigid command-and-control (9).

In the specific area of cholera control, a community-based system dynamics study from Nigeria identified political will, health system resources, and community trust as the main levers for successful implementation—again aligning with the mental-model analysis of the Iceberg Model and the community-centered “choir” of our framework (10). Taken together, these recent studies confirm that the core principles of the Orchestrated Symphony Model are not abstract; they are already emerging as evidence-based priorities for outbreak response in fragile settings. The novel contribution of our framework is to weave these principles into a single, replicable governance architecture with a clear conductor, a shared score, and a rhythm that enables real-time adaptation.

This paper uses the cholera outbreak in Sudan as a case study to operationalize the Orchestrated Symphony Model and to demonstrate its value as both a conceptual framework and an applied model for outbreak response in conflict-affected environments. It aims to achieve three interrelated objectives:

1. To apply systems thinking tools (Iceberg and Biomatrix) to uncover structural, behavioral, and mental models influencing outbreak dynamics.
2. To translate orchestration governance theory into a practical coordination model for epidemic response; and
3. To develop an implementation framework adaptable to fragile and conflict-affected settings.

## 2 Methodology

### 2.1 Study design and approach

This paper employs a qualitative, document-based case study approach to develop and validate the Orchestrated Symphony Framework. A desk review was conducted to synthesize operational and coordination experiences from Sudan’s 2024–2025 cholera response. The orchestral metaphor and its operational definitions were tested with local stakeholders, who readily understood all components. This confirmed the framework’s accessibility and relevance in the Sudan context.

### 2.2 Theoretical foundation: systems thinking and orchestration governance

#### 2.2.1 Systems thinking as a diagnostic and design Lens

System thinking provides a structured approach to understanding complex interactions within health systems. It emphasizes relationships, feedback loops, and patterns rather than isolated events. This analytical perspective is essential for diagnosing the deep-rooted causes of recurrent outbreaks in contexts such as Sudan, where interlocking social, infrastructural, and institutional weaknesses drive epidemics (8–10).

The Iceberg Model serves as the foundational tool for this analysis. It frames problems through four layers: (1) visible events, (2) patterns of behavior over time, (3) underlying structures, and (4) mental models that shape systemic behavior. In Sudan’s cholera outbreak, visible events included widespread cases of acute watery diarrhea, deaths, and overwhelmed treatment centers. The pattern revealed recurring outbreaks across years, indicating chronic vulnerabilities. Structural analysis exposed collapsed water and sanitation systems, delayed response mechanisms, and weak intersectoral coordination. At the deepest level, mental models revealed mistrust between communities, government authorities, and international partners which undermines collective response capacity (11–13).

These levels identify leverage points where interventions can create the most systemic impact: strengthening governance structures, transforming coordination culture, and rebuilding trust. Systems thinking thus forms the analytical core of the Orchestrated Symphony Model, ensuring that orchestration is grounded in an understanding of interdependencies and feedback mechanisms.

Complementing the Iceberg Model, the Biomatrix framework conceptualizes systems through seven components, aim, ethos, structure, process, resources, environment, and governance. Each component provides a lens through which orchestration can be operationalized. For example, “aim” defines the shared strategic goal of zero preventable deaths; “ethos” embodies trust, equity, and collaboration; “structure” maps the orchestration network; “process” defines coordination rhythms; “resources” align human and material assets; “environment” contextualizes external enablers and barriers; and “governance” ensures adaptive leadership. Integrating these elements ensures a holistic, resilient system architecture.

The Orchestrated Symphony Model is proposed as a policy reform and practice improvement framework for cholera outbreak response in conflict-affected settings.

#### 2.2.2 Orchestration governance: from command to conduct

Orchestration governance theory redefines the role of the state in complex systems. Rather than exerting control through command hierarchies, the state acts as an orchestrator—catalyzing, convening, and aligning diverse actors toward a shared objective (6). The IBM Center for the Business of Government first articulated this shift as a movement from “command and control” to “orchestrate and enable” (6).

In the context of Sudan’s cholera outbreak, the Ministry of Health becomes the conductor of a national health orchestra. The outbreak response plan functions as the musical score. Stakeholders, such as federal and state health authorities, humanitarian agencies, NGOs, community organizations, epidemiologists, and WASH workers, serve as instruments. Each actor plays a distinct part but follows the same score and tempo, creating harmony instead of chaos.

This orchestration metaphor captures the essence of adaptive governance in crisis settings. It combines centralized vision with decentralized execution, where coordination replaces hierarchy, feedback replaces rigidity, and learning replaces control. The model’s strength lies in its capacity to accommodate diverse actors within a coherent framework, enabling rapid decision-making and continuous adjustment based on real-time data (14).

During Sudan’s outbreak, the Ministry’s Public Health Emergency Operations Center (PHEOC) embodied aspects of orchestration through real-time data sharing, daily coordination calls, and hybrid (virtual and in-person) meetings (15). However, limited resources, fragmented partner engagement, and delayed preparedness measures exposed the need for a more systematic orchestration model that institutionalizes such coordination mechanisms.

The Orchestrated Symphony Model builds on this experience, proposing a structured yet flexible system for harmonizing actions across sectors and governance levels. It is especially suited to crisis-affected contexts where centralized authority may be weakened and where adaptive, networked collaboration is essential for sustaining health service delivery (6).

### 2.3 Document selection criteria

Documents were purposively selected for their relevance to the outbreak governance (16), coordination processes, and implementation realities. Inclusion criteria were: (i) any accessible documents produced between July 2024 and June 2025; (ii) related to Sudan’s cholera response; (iii) with information on coordination mechanisms, decision-making, field operations, or outcomes; and (iv) and that originates from official sources including Federal or state Ministry of Health, WHO, or partner organizations involved in cholera response. Exclusion criteria were: (i) duplicate documents; (ii) documents with no clear date source, and (iii) unverified media reports.

A total of 19 documents were reviewed, including 3 public health emergency operations centre (PHEOC) reports, 3 field visit reports and 2 investigation reports, 2 state level and 2 locality level response (activities) reports, 3 health cluster meeting minutes outbreak, and the national cholera plan (see Table 1 for more details on targeted contents in each document).

**Table 1:** Summary of documents reviewed and analytical focus.

Type of document reviewed	Analytical focus applied
PHEOC meeting minutes	Identification of coordination and decision-making challenges, patterns, structural constraints, and mental models, as represented by the Iceberg Model, serves as input for Biomatrix mapping.
Field visit reports	Diagnosis of systemic bottlenecks; triangulation of findings; application of the Orchestrated Symphony Model to field experiences.
Outbreak investigation summaries	Validation via triangulation; recognition of recurring patterns, constraints, and adaptive strategies.
Activity documentation from affected localities	Mapping and Analysis of patterns and structures; synthesis for integration of the Biomatrix model; evaluation of orchestration effectiveness.
Supervision mission reports	Utilization of triangulation with additional secondary data; analysis of structural and systemic factors.
Health cluster coordination meeting reports	Identification of governance frameworks; recognition of systemic limitations; incorporation into orchestration model.

This purposive sampling approach allowed focused analysis of materials that best reflected real-time implementation realities and orchestration dynamics.

### 2.4 Analysis

A preliminary analytical framework was developed based on the Iceberg Model (events, patterns, structures, mental models) and the Biomatrix system components (aim, ethos, structure, process, resources, environment, governance). Manual thematic analysis was done based on the above framework. Coding was performed manually by two authors (FA and AI), with disagreements resolved through discussion and consensus with a third author (ME). Recurrent themes were iteratively refined.

Validation was achieved through triangulation with reports from outbreak investigations. Triangulation was achieved by comparing findings across multiple document types and sources.

The stepwise analysis stages were:

1. Systemic diagnosis: using the iceberg model, the analysis identified visible events, recurring patterns, structural constraints, and mental models influencing response effectiveness.
2. Framework integration: insights from the systemic diagnosis were mapped onto the Biomatrix model to construct a comprehensive architecture for orchestration.
3. Model application: the framework was tested against field experiences from Khartoum, White Nile, Gadaref, and Darfur states to evaluate its explanatory and operational utility.

No primary data was collected. Findings are derived exclusively from secondary documentation and interpretive synthesis.

The resulting framework—the Orchestrated Symphony Model—functions as both a conceptual tool and an operational blueprint for managing outbreak coordination in conflict-affected settings. While grounded in the Sudan case, its design allows adaptation to other fragile contexts facing recurrent epidemics.

### 2.5 Researcher positionality and reflexivity

Several authors are affiliated with the Federal Ministry of Health (FMoH) and were directly involved in the cholera response (FA, AI, HA). This insider perspective provided unique access to unpublished operational documents and real-time contextual understanding. However, it also carries potential for bias in document selection and interpretation. To mitigate this, we: (i) included a co-author (ME) from an independent academic institution (University of Geneva) who was not involved in the response; (ii) applied a systematic inclusion/exclusion criterion to document selection; (iii) used framework analysis with explicit coding rules; and (iv) actively searched for disconfirming evidence (e.g., reports of coordination failures). We state that the findings and framework represent the authors’ best interpretation of available evidence and may benefit from independent perspective validation.

## 3 Results

### 3.1 Designing the score: development of the Orchestrated Symphony Framework

The Orchestrated Symphony Framework was conceptualized as a hybrid model that merges systems thinking analysis with orchestration-based governance. It evolved through iterative integration of the *Biomatrix* and *Iceberg* tools, supported by operational documentation from Sudan’s 2024–2025 cholera outbreak. The resulting framework positions health response coordination as a living, adaptive system that requires alignment between vision, structure, processes, resources, environment, and governance.

The model is rooted in the principle of “Think System, Act Orchestra.” This implies that outbreak management is not a static set of technical actions but a continuous process of harmonization across system elements. Each actor functions as a distinct instrument; coordination is the composition that produces a unified performance (see Table 2).

**Table 2:** Secular glossary of orchestral terms used in the framework.

Musical term (metaphor)	Operational term (plain language)
Conductor	Central coordination body (e.g., Ministry of Health, PHEOC)
Score	Strategic response plan (objectives, milestones, indicators, resource map)
Tempo/Rhythm	Operational cadence (e.g., twice-daily coordination calls, weekly review cycles)
Sections (strings, brass, woodwinds, percussion)	Technical pillars (surveillance, case management, risk communication, WASH)
Choir	Community engagement network (volunteers, local leaders, civil society)
Concert hall	Operational environment (security, access, social and political context)
Baton	Governance and accountability mechanism (real-time dashboards, feedback loops)

The framework’s design began with a review of systemic failures evident during Sudan’s outbreak response. Weak preparedness, delayed resource deployment, and limited intersectoral alignment revealed deficiencies in structure and process (17). Simultaneously, fragmented leadership and limited trust reflected governance and ethos challenges. By mapping these issues through the *Biomatrix* model, seven leverage components were identified: aim, ethos, structure, process, resources, environment, and governance.

These seven components were then mapped onto orchestral metaphors to create a practical guide for coordination. For example, the *aim* corresponds to the “conductor’s score,” providing direction and tempo; *ethos* aligns with the musical genre, shaping tone and culture; *structure* forms the orchestra sections, assigning clear roles; *process* provides rhythm and flow; *resources* represent the instruments; *environment* becomes the concert hall; and *governance* embodies the conductor’s leadership.

Each component corresponds to a specific orchestration function and guiding question that can inform outbreak management. This structure ensures that coordination mechanisms are not arbitrary but grounded in systemic logic.

### 3.2 Systems thinking in framework design

**Figure 1:**
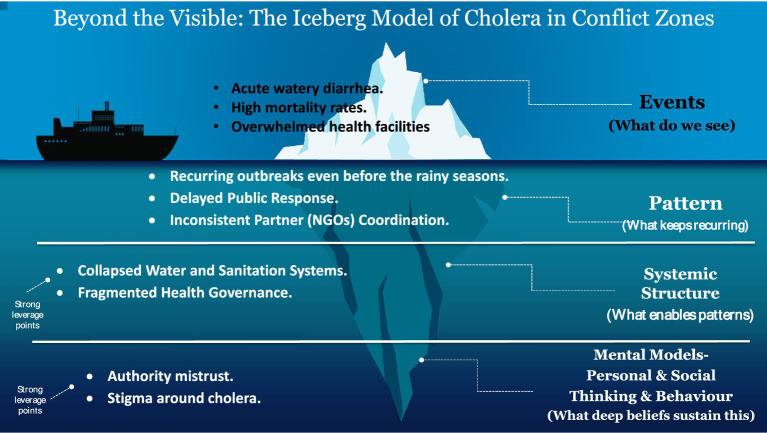
Iceberg model of Sudan’s 2024–2025 cholera outbreak: visible events (cases, deaths), patterns (seasonal peaks, delays), structures (collapsed WASH, weak coordination), and mental models (mistrust), shifting leverage from events to deeper layers.

The systems thinking approach provided the analytical foundation for identifying underlying causes of Sudan’s recurrent cholera outbreaks. Using the Iceberg model (Figure 1), visible events—such as acute watery diarrhea cases, deaths, and congested health facilities—were analyzed alongside recurring patterns, including seasonal peaks and delays in response. Structural analysis revealed systemic issues such as the destruction of water and sanitation networks, fragmented coordination between sectors, and resource mismanagement.

Deeper analysis at the level of mental models exposed a prevailing atmosphere of mistrust: communities perceived cholera as a death sentence; partners doubted national capacity; and authorities feared international oversight.

The systems perspective emphasized intervention at the levels of structures and mental models. Thus, the framework focuses on governance reform, coordination culture, and community trust-building.

### 3.3 From systems thinking to orchestration

The Orchestrated Symphony Framework translates systems thinking insights into orchestration functions. Each component of the Biomatrix framework aligns with an orchestral role:

- Aims (vision): the score defines the ultimate objective, e.g., zero preventable cholera deaths within 60 days.
- Ethos (culture): the genre sets the tone of collaboration, trust, and equity across actors.
- Structure: The orchestra sections define clear responsibilities for surveillance, case management, WASH, and logistics.
- Process: the rhythm represents the sequence and timing of interventions, such as daily coordination briefings and tasking cycles.
- Resources: instruments represent the assets mobilized—supplies, funding, and human resources.
- Environment: the concert hall represents the operational context, including security, social factors, and access constraints.
- Governance: the conductor provides leadership, alignment, and accountability.

### 3.4 Conceptual framework: the dual-dimensional logic of the Orchestrated Symphony Model

**Figure 2:**
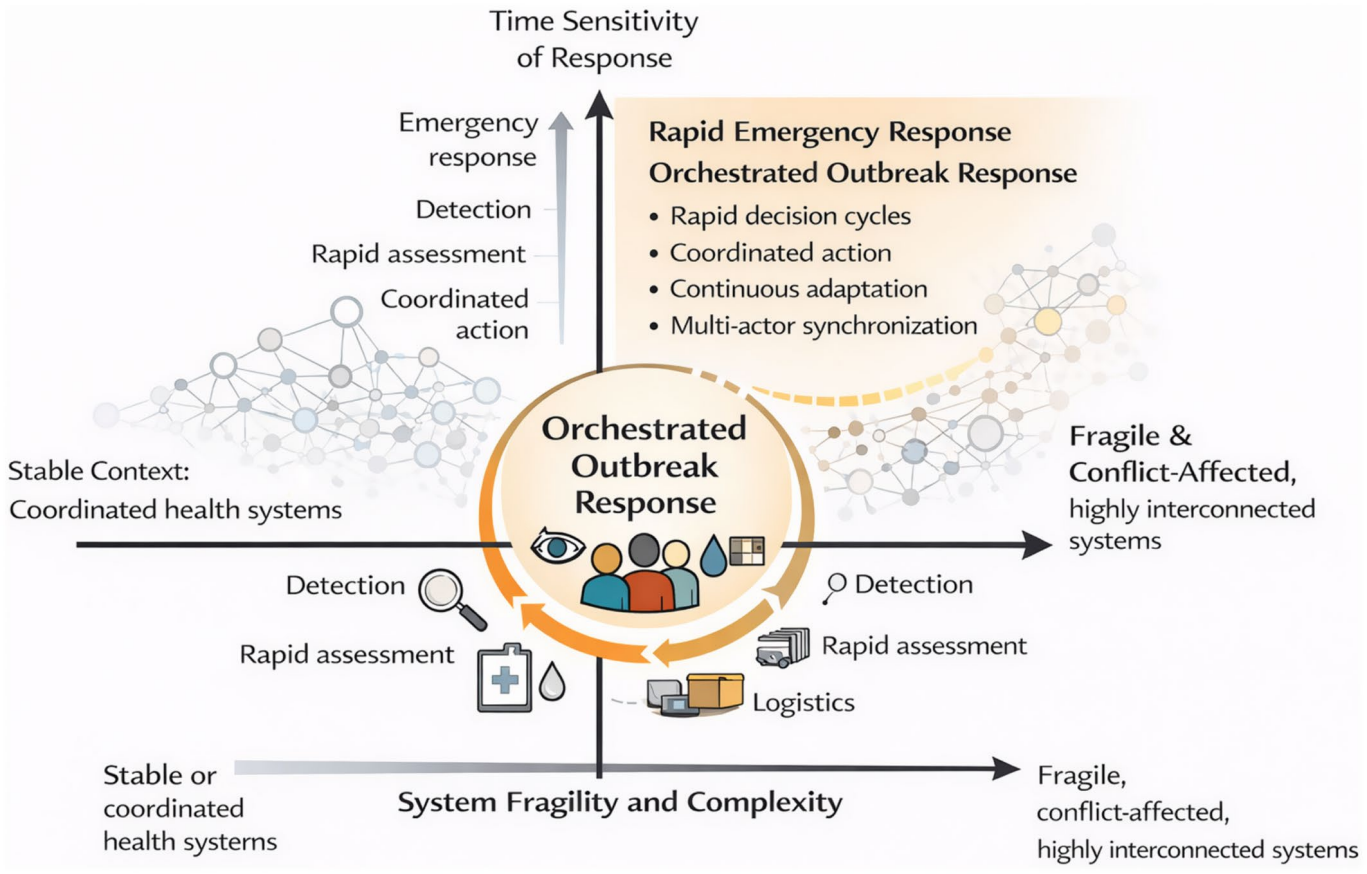
The orchestrated symphony model of epidemic response. The model conceptualizes outbreak response in fragile settings through two interacting dimensions: system fragility and complexity (horizontal axis) and time sensitivity of response (vertical axis). Their intersection highlights the need for orchestrated, adaptive coordination among multiple actors to manage epidemics in rapidly changing conflict-affected environments. “This figure was created by the authors in Microsoft PowerPoint and visually enhanced using the generative AI tool (ChatGPT)”.

**Figure 3:**
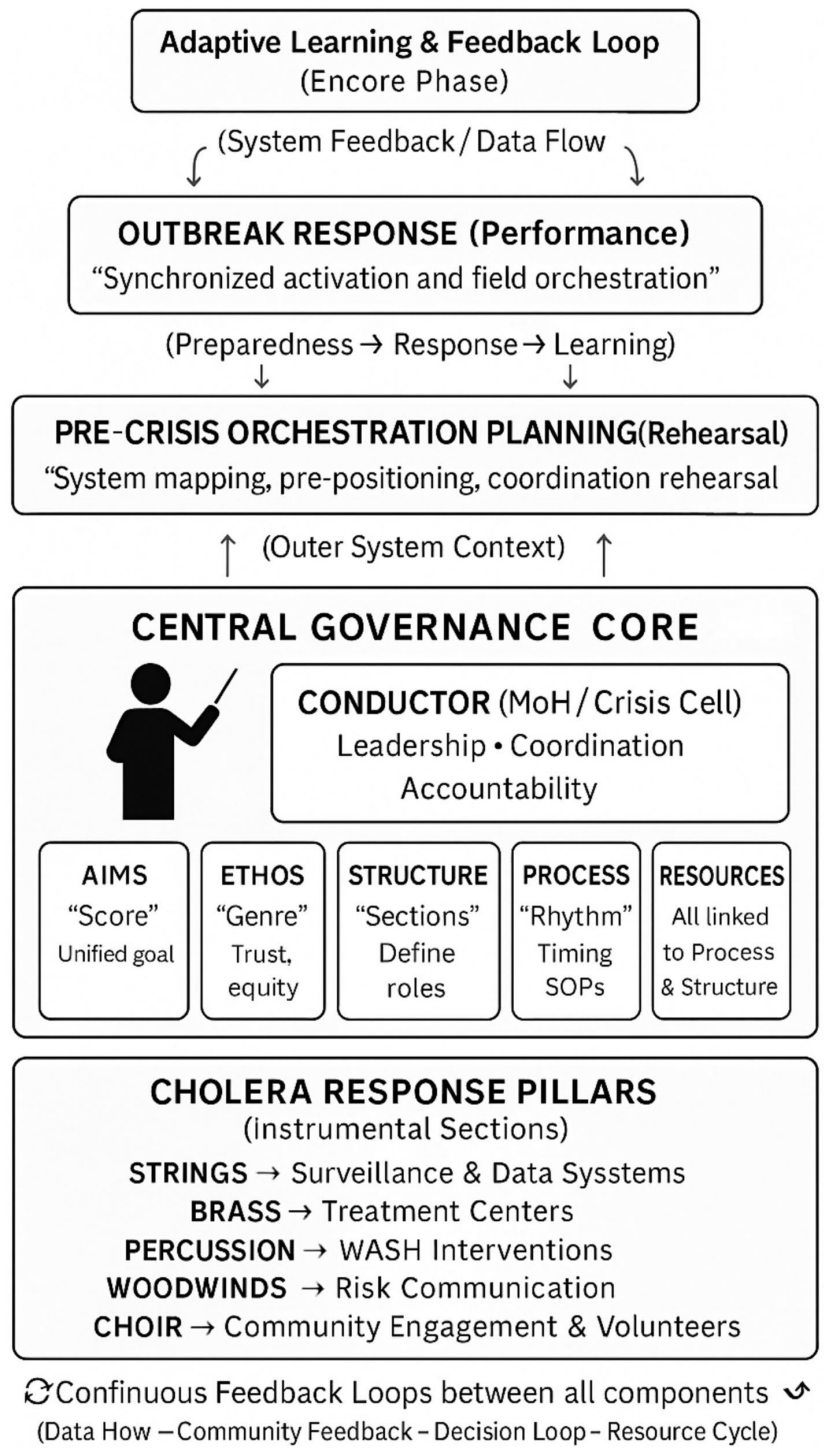
The orchestrated symphony framework mapping seven Biomatrix components (aim, ethos, structure, process, resources, environment, governance) to orchestral roles (conductor, score, sections, rhythm, instruments, concert hall, baton) for coordinated outbreak response in fragile settings.

The Orchestrated Symphony Model conceptualizes epidemic response in fragile, conflict-affected settings through a dual-dimensional framework that integrates system complexity with time sensitivity (Figure 2). The model therefore combines two interdependent dimensions: a horizontal dimension representing the rapidly changing context, where multiple systemic factors interact and shape outbreak dynamics, and a vertical dimension representing the time-sensitive response, which requires rapid assessment, synchronized action, and continuous adaptation (see Figure 3).

### 3.5 Snapshot logic: timely, interconnected sequenced and synchronized operations

A defining feature of the model is the concept of “snapshot operations,” where assessment, diagnosis, intervention, and outcome evaluation occur in parallel rather than in sequence. Snapshot operations create an adaptive rhythm that enables rapid correction, synchronized activation of response pillars, and real-time learning (Table 3).

**Table 3:** The Orchestrated Symphony Framework: alignment of Biomatrix system elements with orchestration roles, administrative actions, and cholera response applications in conflict-affected Sudan.

Biomatrix element	Definition	Orchestra role (metaphor)	Orchestration role (practical function)	Administrative action	Guiding questions	Cholera-specific example
Aims (vision)	The outcome the system strives to achieve; creates direction and focus for collective action.	Conductor’s Score	Sets unified purpose and tempo for all actors.	Define the national outbreak goal and align partners around it.	- Which health outcomes are most urgent? - Do we target containment, prevention, or resilience—or all three? - How can short-term control be balanced with long-term recovery amid war?	“Zero preventable cholera deaths in 60 days”
Ethos (culture)	The shared values, norms, and beliefs that shape behavior and trust within the system.	The musical Genre	Builds coherence and trust; defines the ethical tone of collaboration.	Promote trust, equity, transparency, and urgency across all response levels	- Which core values must guide our response? - How can we preserve dignity and equity under conflict conditions? - How does transparency reinforce trust?	Community ownership, dignity, and equity integrated into all response activities.
Structure	The organizational anatomy of the system; defines relationships and responsibilities.	Orchestra Sections	Creates the networked architecture of response actors.	Establish orchestration cells, define cluster leads, clarify mandates.	- Who leads key domains (governance, logistics, case management, WASH)? - How can roles be clarified amid fluid conflict settings? - What existing structures (MoH, NGOs, local leaders) can be leveraged? and local leaders?	Crisis Management unit/Orchestration coordinating MoH, UN, NGOs, CSOs, WASH, other government bodies, community
Process	The sequence and rhythm of coordinated actions for service delivery.	The Rhythm	Synchronizes workflows across teams and levels.	Develop SOPs, daily tasking cycles, and AM/PM coordination briefings.	- How can implementation stay in sync across actors and regions? - Which processes require standardization (e.g., reporting, case management)? - How can real-time data guide immediate adjustments?	Surveillance. case management, Cholera Treatment Centers Mobile clinics, contact tracing WASH campaigns
Resources	The material, financial, and human assets that enable orchestration.	Musicians & Instruments	Aligns and deploys resources efficiently and equitably.	Mobilize personnel, logistics, funding, and local assets.	- What essential resources (supplies, personnel, funds) are available? - How can gaps be bridged rapidly? - How can social capital (volunteers, diaspora) be leveraged? - How do we balance emergency response with health system resilience?	Domestic and Humanitarian resources, rapid response teams, OCV stockpiles, diaspora mobilization, local volunteers’ networks.
Environment	The physical, political, and cultural context that shapes system performance.	The Concert Hall	Navigates contextual barriers and enablers.	Map access constraints, stakeholder dynamics, and community perceptions.	- What contextual factors (security, access, social trust) shape the response? - How does war affect mobility and logistics? - Which community structures can bridge communication gaps?	Mapping safe zones, humanitarian corridors, adapting to conflict sensitivity
Governance	The leadership, regulation, and adaptive monitoring that sustain system alignment.	The Baton	Directs tempo, enforces discipline, and ensures harmony across the system.	Activate adaptive coordination mechanisms, define accountability, monitor progress.	- How do we regulate and monitor progress while adapting to rising cases? - Which governance mechanisms ensure agility and accountability? - How can leadership remain responsive under uncertainty?	Real-time shared dashboards, decentralized leadership, task shifting protocols, public reporting

The seven components and their definitions are drawn from the Biomatrix systems framework. The orchestral mapping, administrative actions and cholera-specific examples are derived from the authors’ document analysis of Sudan’s 2024–2025 outbreak response (see Methods).

### 3.6 Application of cholera response pillars in the orchestrated symphony approach = playing the symphony

#### 3.6.1 Application of the framework: the Sudan cholera case study

The 2024–2025 epidemic unfolded amid extreme fragility, where centralized command systems had weakened, and local capacities varied greatly among states (15–18). Applying orchestration principles allowed identification of how coordinated action—or its absence—shaped outbreak trajectories.

##### 3.6.1.1 The conductor: Ministry of Health and the national crisis cell

During the outbreak, the Federal Ministry of Health (FMoH) operated through its Public Health Emergency Operations Center (PHEOC), which acted as the central conductor. The PHEOC coordinated the overall response through real-time surveillance updates, situation reports, and regular virtual and in-person meetings. This structure partially reflected orchestration governance: centralized direction with decentralized implementation.

Resource constraints included insufficient preparedness budgets, which delayed treatment center activation and supply prepositioning. Reduced partner engagement led to weakened field-level coordination.

##### 3.6.1.2 The strings: surveillance and data systems

Surveillance functions provided data-driven decision-making. Early warning systems detected spikes in acute watery diarrhea in seven localities, triggering alerts; however, response delays occurred due to resource shortages and communication gaps between local and federal levels (19).

In Khartoum, surveillance data identified hotspots—particularly Karari and Jabel Awlia—where mortality was highest. Using these data, the vaccination committee conducted a reactive oral cholera vaccine (OCV) campaign targeting 12 administrative units in five localities (15). Over 2.2 million people were vaccinated, achieving 85% coverage despite conflict constraints. Weekly cases declined following the campaign (19).

##### 3.6.1.3 The brass: treatment centers and mobile clinics

Treatment facilities included mobile clinics and cholera treatment centers (CTCs). In Umbada locality, late access due to security barriers delayed response. Once the area stabilized, the FMoH mobilized domestic resources to reopen Umbada Hospital as a CTC. This facility served patients and reduced occupancy at other centers.

##### 3.6.1.4 The percussion: WASH teams

Water, sanitation, and hygiene (WASH) teams conducted environmental interventions such as chlorination campaigns and rehabilitation of water stations. In Khartoum, WASH partners coordinated with the FMoH to conduct large-scale chlorination of water sources.

##### 3.6.1.5 The woodwinds: risk communication and community engagement

Risk communication (RC) activities during the Sudan outbreak were limited in scope and funding. Messaging focused mainly on discouraging unsafe water use. Integration of RC into vaccination and WASH campaigns was documented.

##### 3.6.1.6 The choir: volunteers and community leaders

Volunteers and community leaders were mobilized. In White Nile State, students from local universities volunteered for active case finding, risk communication, and chlorine distribution.

#### 3.6.2 Subnational case illustrations

##### 3.6.2.1 White Nile and Khartoum: coordinated harmony

**Figure 4:**
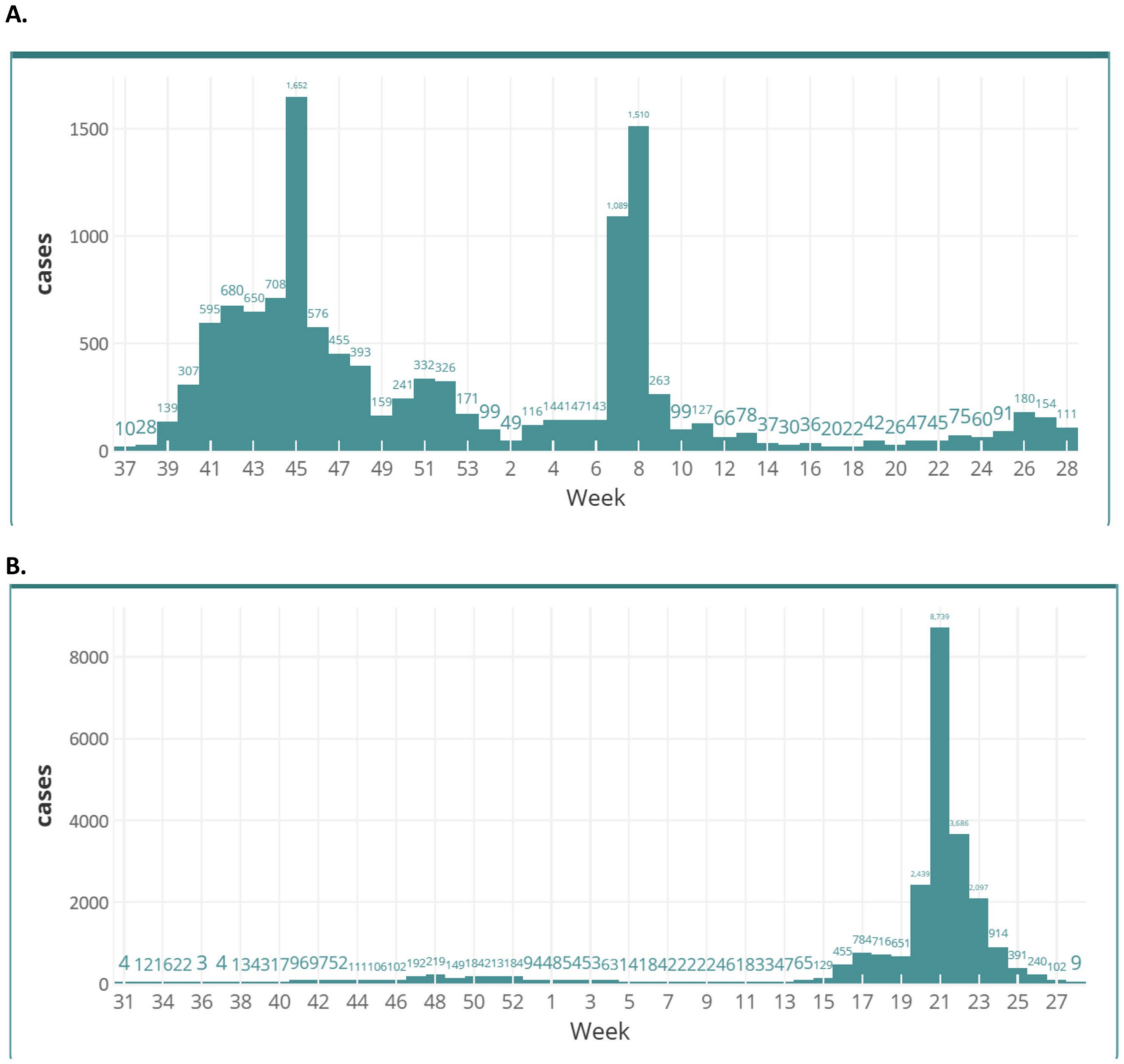
Epidemic curves showing the remarkable drop in weekly reported cholera cases after successful well-coordinated efforts in White Nile **(A)**, and Khartoum **(B)**.

In White Nile and Khartoum states, Ministry of Health’s mobilized of resources, coordinated partners, and prioritized life-saving interventions and led integrated efforts between health and WASH sectors that included establishing treatment centers, repositioning medical supplies, and conducting OCV campaigns. Epidemiological curves showed declines (Figure 4) in weekly cases following these interventions (20).

##### 3.6.2.2 Gadaref: the dissonance of fragmentation

In Gadaref State, resources were available and partners were active, but Interventions were delayed, overlapping, or misaligned. Internally displaced populations remained affected.

##### 3.6.2.3 Darfur: the hybrid orchestration model

In Darfur, federal coordination was maintained through the SMoH, while partners such as WHO, UNICEF and Médecins Sans Frontières (MSF) conducted field operations. Actions remained aligned with the national outbreak plan (see Table 4).

**Table 4:** Operational implementation: stakeholder roles with performance indicators for cholera response.

Orchestra role (metaphor)	Stakeholder/function	Key functions in response	Performance indicators (target by mid-2026)
Conductor	Federal & State Ministry of Health (crisis cell/orchestration cell)	Overall leadership, coordination, and strategic direction	Response lag: < 7 days from alert to action Governance: Twice-daily AM/PM orchestration calls; updated outbreak plan
Strings	Surveillance & Data Reporting Teams	Early detection, case confirmation, real-time reporting	Reporting coverage: ≥ 85% facility-based; ≥ 30% increase in community alerts
Brass	Cholera Treatment Centers/Mobile Clinics	Case management, patient care, and referral	CFR: < 1% Bed Occupancy: < 85% surge capacity
Percussion	WASH Partners (chlorination, latrines)	Environmental interventions, safe water supply, sanitation	WASH reach: ≥ 80% coverage within 7 days of first confirmed case
Woodwinds	Risk Communication & Community Engagement Teams	Public messaging, rumor management, mobilization	Community engagement: ≥ 80% of households reached with actionable messages
Choir	Volunteers & Community Leaders	Grassroots mobilization, trust building, local implementation	OCV coverage: ≥ 30% in hotspot areas (preventive campaigns)

Indicators and targets are derived from WHO/GTFCC benchmarks, the 7-1-7 framework, and the national cholera plan for OCV and other targets.

##### 3.6.2.4 Applying cholera response pillars in the orchestrated symphony approach

The Orchestrated Symphony Framework aligns with the established cholera response pillars, surveillance, case management, WASH, risk communication, and coordination.

- Surveillance (strings): generates data-driven rhythm for decision-making.
- Case management (brass): provides visibility and strength at critical intervention points.
- WASH (percussion): Drives the operational pulse of prevention.
- Risk communication (woodwinds): adjusts tone to maintain community trust.
- Community engagement (choir): amplifies resilience through collective participation.

## 4 Discussion

### 4.1 Looking ahead: cholera in the next two years, from fragility to foresight

Cholera remains a persistent risk in Sudan given the interplay of several vulnerabilities: infrastructure damage, including WASH, displacement, high vulnerability to flooding, limited access to healthcare, limited resources and disrupted governance (21). Therefore, it is not a question whether will cholera outbreaks occur? but instead, how severe and widespread the outbreaks will be. The experience from the 2023–2024 outbreaks, provides insightful lessons for redesigning a predictive, coordinated, community-driven, and resilient response.

The next two years represent an important window for policy reform, where cholera response must be shifted from reactive interventions to proactive containment through strategic orchestration. This transition requires evidence-based planning, sustainable investments in system redesign, and governance reforms and resources for early detection, risk mapping, priority-based preparedness, rapid resource mobilization, decentralized coordination and community empowerment.

The 2024–2025 cholera outbreak in Sudan revealed the limitations of traditional outbreak management within fragile and conflict-affected environments. Centralized command structures, while efficient in stable contexts, often falter amid systemic fragility, displacement, and resource scarcity. The Orchestrated Symphony Framework addresses this gap by repositioning coordination as a living, adaptive system that synchronizes multiple actors toward a shared vision.

This model reframes outbreak governance as a collective performance in which success depends on both technical precision and relational harmony. Through the integration of systems thinking and orchestration governance, health authorities can identify structural leverage points, design adaptive processes, and maintain coherence across decentralized teams.

#### 4.1.1 Interpreting subnational experiences

The subnational case illustrations from White Nile, Khartoum, Gadaref, and Darfur—described in Results—reveal a consistent pattern. Where coordination mechanisms were synchronized with a clear conductor and shared rhythm (White Nile and Khartoum after orchestration was activated), case declines followed more rapidly. This suggests that orchestration produced measurable improvements even when implementation was delayed. In White Nile and Khartoum, the Ministry of Health’s governance and leadership facilitated mobilization of resources, coordination of partners, and prioritization of life-saving interventions. The sharp declines in weekly cases following integrated efforts (Figure 4) demonstrate the potential of well-orchestrated, data-guided action.

By contrast, in Gadaref State, resources were available and partners were active, yet leadership weaknesses and coordination gaps hindered timely action. The lack of harmonization produced operational inefficiencies, leaving internally displaced populations vulnerable. This scenario illustrates the consequences of weak orchestration—an orchestra without a conductor, with instruments playing out of tune (6). The dissonance observed in Gadaref underscores that resource availability alone is insufficient without systematic coordination.

In Darfur, where government structures were severely constrained by insecurity, an alternative hybrid orchestration emerged. Federal coordination was maintained through the SMoH, while partners such as WHO and MSF conducted field operations. Despite decentralization, actions remained aligned with the national outbreak plan. This adaptive conducting demonstrates how coordination can be sustained even in inaccessible regions when orchestration principles are applied flexibly.

Regarding risk communication and community engagement, the Sudan experience highlighted the need for elevating RC as an independent orchestration pillar. Messaging focused mainly on discouraging unsafe water use, but without viable alternatives, communities were reluctant to comply. When RC was integrated into vaccination and WASH campaigns, coverage improved—yet communication risked being reduced to a secondary activity. Maintaining trust and behavioral alignment requires dedicated orchestration of community engagement, not merely add-on messaging. The mobilization of volunteers and community leaders in White Nile State—students conducting active case finding, risk communication, and chlorine distribution—embodied the ethos of orchestration: community-centered, decentralized, and harmonized action. This bottom-up engagement provides a replicable model for other regions.

### 4.2 Two diverging paths for cholera response

The analysis of Sudan’s outbreak trajectories suggests two possible futures for epidemic management in fragile settings.

#### 4.2.1 Scenario 1: business as usual — rising burden, waning trust

In the absence of major shifts in outbreak preparedness and governance, Sudan will likely continue to experience frequent and large-scale cholera outbreaks. Looking at the recent trends in the first wave (Aug 2023–May 2024) and the second wave (July 2024–July 2025), the epidemiological indicators multiplied 10 times; Incidence increased from 35/100,000 to 231/100,000; the CFR remained significantly high exceeding the WHO benchmark of 1%, with 2.4–2.6%; the geographical areas affected expanded from 80 localities in 12 states to 109 localities in 17 states. Delay in response, whether due to coordination or logistical issues or poor community engagement, prolongs outbreaks, increases the cost of response and worsens the health outcomes (22).

Without reform, Sudan’s cholera response risks perpetuating fragmentation, requiring greater resources for each new outbreak, generating higher mortality, and eroding public trust.

### 4.3 Scenario 2: strategic reform — containment, resilience, and recovery

By contrast, institutionalizing orchestration governance can transform outbreak management into a proactive, community-driven, and data-informed process. The Orchestrated Symphony Framework offers a roadmap for such reform. Key strategies include Strengthening of localized early warning systems. Conducting multilevel outbreak simulations to enhance preparedness. Establishing community engagement platforms linked to federal and state monitoring. Investing in integrated WASH–health programming. Adopting decentralized leadership cells capable of rapid deployment. Through orchestration, Sudan could significantly reduce morbidity and mortality within two years, achieving measurable improvements in response time and coverage.

Proposed indicators under a reformed orchestration-based approach a case fatality ratio below 1%, this is based on the WHO benchmark when the response is optimal and also aligns with the global road map of reducing cholera mortality by 90% (23). Response lag 7 days or less, which is based on the internationally recognized 7–1-7 metrics for outbreak response (24). Additional targets include at least, 80% WASH intervention reach within one week of the first confirmed case, and a 30% increase in community-level surveillance reporting, this aims to increase the detection capacity particularly at the community level. Also, a target on preventative OCV overage was set based on the updated country national cholera plan 2026–2030, where the priority hotspot areas are targeted (25) see Tables 5, 6 for complete target benchmarks.

**Table 5:** Expected epidemiological indicators—with reform-based preparedness.

Indicator	Target
CFR	< 1%
Time from Alert to Response	5–7 days
WASH Intervention Reach	>80% within 7 days of first confirmed case
Surveillance and reporting	community reporting increased by >30% from baseline, and facility-based reporting 85 -95%
OCV Coverage	>30% coverage of the hotspot areas with preventive OCV campaigns

Indicators and targets are derived from WHO/GTFCC benchmarks, the 7-1-7 framework, and the national cholera plan for OCV and other targets.

**Table 6:** Indicators of progress: what to measure, what to aim for.

Indicator	2024–2025 observed	Target by mid-2026
CFR	2.5%	< 1%
Response lag	12–21 days	< 7 days
WASH reach	< 50%	≥80%
Community alerts	Low/Delayed	Real-time, first-hand
Preventative OCV coverage	0%	≥30%

Indicators and targets are derived from WHO/GTFCC benchmarks, the 7-1-7 framework, and the national cholera plan for OCV and other targets.

### 4.4 The implementation model: conducting in conflict

The Orchestrated Symphony Framework translates into a three-phase implementation model that can guide outbreak management in fragile contexts. Each phase corresponds to a stage in the symphonic process: prelude, performance, and adaptation.

#### 4.4.1 Phase 1: pre-crisis orchestration planning (preparedness)

Preparedness functions as the rehearsal. systemic mapping through iceberg and biomatrix analyses identifies structural weaknesses and leverage points. The Ministry of Health, acting as conductor, establishes orchestration cells at federal and state levels. These cells define roles, develop coordination protocols, and plan simulation exercises. Pre-positioning of supplies and pre-defined communication channels ensure readiness to “perform” when the crisis begins.

#### 4.4.2 Phase 2: rapid deployment at outbreak onset (performance)

Once an outbreak is detected, orchestration moves from planning to action. The crisis cell becomes the operational conductor, initiating synchronized activation of all orchestral sections: surveillance (strings), treatment centers (brass), WASH (percussion), risk communication (woodwinds), and community engagement (choir). Each section operates autonomously yet in rhythm with others. Real-time dashboards serve as the musical score, ensuring that every actor plays the right note at the right time.

#### 4.4.3 Phase 3: real-time adaptive conducting (control and containment)

The final phase emphasizes continuous feedback and adaptive leadership. Twice-daily orchestration meetings (“AM/PM tempo sessions”) evaluate progress, identify dissonance, and adjust strategies. Decisions are guided by live surveillance data and community feedback, transforming governance into an iterative, learning-based process. This adaptive conducting enables rapid correction of misalignments and ensures that the response remains harmonized despite shifting field conditions.

Together, these phases form a cyclical process—preparedness feeding into response, and response informing reform. The system, like an orchestra, improves with each rehearsal and performance, building institutional memory and resilience.

#### 4.4.4 Bridging silence and sound: insights from the model

The integration of systems thinking and orchestration governance offers a conceptual bridge between analysis and action. Systems thinking diagnoses the structural and behavioral underpinnings of fragility; orchestration governance translates those insights into practice. The result is a governance framework that is neither rigidly hierarchical nor loosely networked but dynamically coordinated.

The Orchestrated Symphony Framework thus bridges three persistent divides in health emergency management:

1. Between hierarchical control and collaborative coordination.
2. Between isolated technical interventions and integrated multisectoral strategies.
3. Between reactive crisis management and proactive system resilience.

This paradigm aligns closely with the World Health Organization’s Health System Building Blocks, reinterpreted through the lens of orchestration (see Table 7).

**Table 7:** Transitioning from fragmented response to orchestrated governance (applied to WHO Health system building blocks).

Building block	From	To (orchestration model)
Governance & leadership (coordination)	Centralized command/control *Ad hoc* cluster meetings	Orchestration Governance; Twice-daily “AM/PM tempo” orchestration calls with feedback realignment
Health workforce	Fragmented responders with unclear roles	Trained decentralized response teams funded by domestic resources ‘instrumental parts (surveillance, case management, WASH, etc.)
Health information systems	Vertical reporting and late data sharing	Real-time, shared dashboards and daily field-to-lead feedback loops
Service delivery	Static, top-down service delivery	Flexible, delivered at community and local levels with coordination rhythm
Medical products	Delayed, siloed procurement and stockouts	Coordinated logistics cell with demand–supply harmonization
Health financing	Donor-fragmented funding streams	Pooled, agile funding tied to orchestration nodes and performance
Community engagement	Messaging as one-way broadcast	Community as “choir”: co-design, co-lead, monitor feedback
At glance “From fragmentation to flow. From silos to symphony. From control to coordination.”		

Under the orchestration model, leadership and governance shift from command-and-control to adaptive coordination. The health workforce becomes a decentralized network of trained responders aligned under a unified direction. Information systems evolve into real-time dashboards, replacing vertical reporting with feedback loops. Service delivery transitions from static to flexible, community-based operations. Procurement and logistics transform from siloed processes to synchronized supply orchestration. Financing becomes pooled and performance-based, while community engagement moves from one-way communication to co-leadership.

This systemic transformation can be summarized as a movement “from fragmentation to flow, from silos to symphony, from control to coordination”.

#### 4.4.5 Looking ahead: cholera and the next two years

The risk of future cholera outbreaks in Sudan remains high due to structural vulnerabilities—damaged WASH infrastructure, displacement, and disrupted governance. The question is not whether outbreaks will recur, but how severe they will be. The coming two years present a critical window to institutionalize orchestration-based reforms.

A forward-looking strategy must prioritize the integration of orchestration into national epidemic preparedness frameworks. This includes establishing a permanent orchestration unit within the Ministry of Health, developing a national outbreak “score” adaptable to different hazards, and ensuring continuous rehearsal through simulation exercises.

At the community level, engagement must transition from passive information sharing to co-designed action. Community leaders, volunteers, and civil society groups should be embedded as integral components of the orchestra, ensuring that response rhythms resonate with local realities.

By operationalizing orchestration, Sudan can shift from a reactive emergency posture to a predictive, resilient system capable of managing epidemics amid conflict and recovery.

### 4.5 Limitations

The limitations of this study include the following: first, it is document-based, using secondary operational data. Second, author affiliation with the Ministry of Health, although mitigated by systematic selection, dual coding, and an independent co-author, full objectivity is not guaranteed. Lastly, the Orchestrated Symphony Framework remains conceptual; its performance indicators are proposed targets, not validated measures of the 2024–2025 response. Given these limitations, generalizability beyond Sudan requires prospective validation.

## 5 Conclusion

The 2024–2025 cholera outbreak in Sudan represents both a tragedy and a lesson in systemic fragility. It demonstrates the insufficiency of traditional outbreak response mechanisms that rely on centralized command, sectoral silos, and fragmented coordination. The Orchestrated Symphony Framework offers an alternative scientifically grounded, conceptually coherent, and operationally viable model for managing health emergencies in fragile and conflict-affected settings.

By integrating systems thinking and orchestration governance, this model transforms outbreak response into a dynamic, adaptive, and collaborative process. It emphasizes that the Ministry of Health must act as conductor, guiding a network of actors toward synchronized action. Each actor, like a musician in a symphony, contributes to a collective performance defined by shared goals, mutual trust, and real-time learning.

This framework not only explains why the Sudan cholera outbreak unfolded as it did but also provides a roadmap for preventing future crises. It calls on national and international health actors to move beyond fragmented interventions and embrace orchestration as a new standard of practice.

### 5.1 Policy imperative: from lessons to legacy

The lessons from Sudan’s outbreak must be translated into institutional reform. The immediate priority is to pilot and simulate the Orchestrated Symphony Framework through real-world exercises. Integrating this model into national preparedness plans will ensure readiness for future crises.

## Data Availability

The original contributions presented in the study are included in the article/supplementary material, further inquiries can be directed to the corresponding author.
